# Association of sleep disturbances within 4 weeks prior to incident acute myocardial infarction and long-term survival in male and female patients: an observational study from the MONICA/KORA Myocardial Infarction Registry

**DOI:** 10.1186/s12872-018-0969-z

**Published:** 2018-12-13

**Authors:** Franziska Nairz, Christa Meisinger, Inge Kirchberger, Margit Heier, Christian Thilo, Bernhard Kuch, Annette Peters, Ute Amann

**Affiliations:** 1 Central Hospital of Augsburg, MONICA/KORA Myocardial Infarction Registry, Augsburg, Germany; 2 Helmholtz Zentrum München, German Research Center for Environmental Health (GmbH), Institute of Epidemiology II, Neuherberg, Germany; 3 Institute for Medical Informatics, Biometry and Epidemiology (IBE), LMU Munich, Munich, Germany; 4 Ludwig-Maximilians-Universität München, UNIKA-T, Augsburg, Germany; 5 Central Hospital of Augsburg, Department of Internal Medicine I – Cardiology, Augsburg, Germany; 6 Hospital of Nördlingen, Department of Internal Medicine/Cardiology, Nördlingen, Germany; 7 KORA-Herzinfarktregister im Klinikum Augsburg/Helmholtz Zentrum München, Stenglinstr. 2, 86156 Augsburg, Germany

**Keywords:** Sleep disturbance, Myocardial infarction, Long-term mortality, Sex differences

## Abstract

**Background:**

Sleep-related investigations in acute myocardial infarction (AMI) patients are rare. The aim of this study was to examine sex-specific associations of patient-reported sleep disturbances within 4 weeks before AMI and long-term survival.

**Methods:**

From a German population-based, regional AMI registry, 2511 men and 828 women, aged 28–74 years, hospitalized with a first-time AMI between 2000 and 2008 and still alive after 28 days, were included in the study (end of follow-up: 12/2011). Frequency of any sleep disturbances within 4 weeks before AMI was inquired by a 6-categorical item summarized to ‘never’, ‘sometimes’ and ‘nightly’. Cox regression models were calculated.

**Results:**

Over the median follow-up time of 6.1 years (IQR: 4.1) sleep disturbances were reported by 32.3% of male and 48.4% of female patients. During the observation period, 318 men (12.7%) and 131 women (15.8%) died. Men who ‘sometimes’ had sleep disturbances showed a 56% increased mortality risk compared to those without complaints in an age-adjusted model (HR 1.56; 95%-CI 1.21–2.00). Additional adjustment for confounding variables attenuated the effect to 1.40 (95%-CI 1.08–1.81). Corresponding HRs among women were 0.97 (95%-CI 0.65–1.44) and 0.99 (95%-CI 0.66–1.49). HRs for patients with nightly sleep disturbances did not suggest any association for both sexes.

**Conclusions:**

Our study found that nightly sleep disturbances have no influence on long-term survival in male and female AMI patients. Contrary to women, men who reported sometimes sleep disturbances had a higher mortality. Further investigations on this topic taking into account the role of obstructive sleep apnoea are needed.

## Background

Sleep disturbances rank as a relevant public health problem in modern society and are expected to increase in the future for several reasons, such as rising job demands combined with 24/7 service availability, inappropriate use of internet or mobile phones at night and ageing populations [1]. Already, about one third of the general population is affected by sleep disturbances in terms of difficulties initiating sleep, maintaining sleep or non-restorative sleep [2]. Sex differences regarding the prevalence of reported insomnia symptoms were shown in previous work and resulted in an estimated female/male ratio of 1.4 which further increased with age [2, 3]. Due to its regenerative function, sleep influences both physical and mental health outcomes. Research in the last decades provided increasing evidence for sleep disturbances - with respect to both sleep quality and quantity - as a risk factor for cardiovascular disease (CVD), cardiac death and all-cause mortality [4–7].

While healthy populations were extensively studied in this context, sleep-related investigations in acute myocardial infarction (AMI) patients are rare. However, previous results indicated that sleep complaints might be associated with disease progression and worse survival prognosis after AMI. Heavy snoring, a proxy for obstructive sleep apnea, was shown to precipitate progression of atherosclerosis in women with AMI or unstable angina pectoris [8] and to be associated with an increased case-fatality in AMI patients [9]. The association between obstructive sleep apnea and risk of CV events and death has been well established in earlier studies [7, 10]. However, recent findings question the impact of obstructive sleep apnea on CV outcomes [11]. Shah et al. [11] suggests a potential cardioprotective effect of obstructive sleep apnea in AMI patients due to ischemic preconditioning. Regarding sleep duration, patients with an acute coronary syndrome who slept less than 7 h per night in the month after the event had an 50% elevated 1-year risk of acute coronary syndrome recurrence and mortality compared to acute coronary syndrome patients who reported longer sleep duration [12]. To our knowledge, only two studies [13, 14] investigated the associations between difficulties initiating sleep or disturbed sleep, respectively, and long-term outcomes in AMI patients. While Condén & Rosenblad [13] followed up recent AMI cases for all-cause mortality without distinction between patients’ sex for the analyses, Clark et al. [14] investigated a composite outcome of incidence of or death from recurrent cardiovascular events during a 10-year follow-up and additionally used data originated from the early 90s.

Hence, the aim of our study was to examine the association of patient-reported sleep disturbances within 4 weeks before incident myocardial infarction (MI) and long-term survival, separately for men and women, using data from the percutaneous coronary intervention era. Sleep disturbances in this study were not regarded as a proxy for any sleep disorder but as a complaint perceived by patients which may have an indicative value for their long-term prognosis.

## Methods

Data for the present observational study stemmed from the population-based MONICA/KORA MI registry in Augsburg (Southern Bavaria, Germany). The study area comprised a total of about 600,000 inhabitants and eight cooperating hospitals. From the beginning in 1984, all cases of coronary deaths and non-fatal MIs of the study population, aged 25–74 years and resident in the study area, have been continuously documented in the registry. Methods of case identification, diagnostic classification of events, and data quality control have been described in detail elsewhere [15, 16]. Briefly, patients hospitalized for AMI and having survived for at least 24 h after hospitalization were interviewed by trained study nurses after transfer from the intensive care unit. They collected information on socio-demographic characteristics, cardiovascular risk factors, medical history of MI, stroke, comorbidities and information on the acute event using a standardized questionnaire. Additional data on AMI characteristics, treatment, in-hospital complications, comorbidities, vital status and discharge medication were provided by medical chart review.

**Fig. 1:**
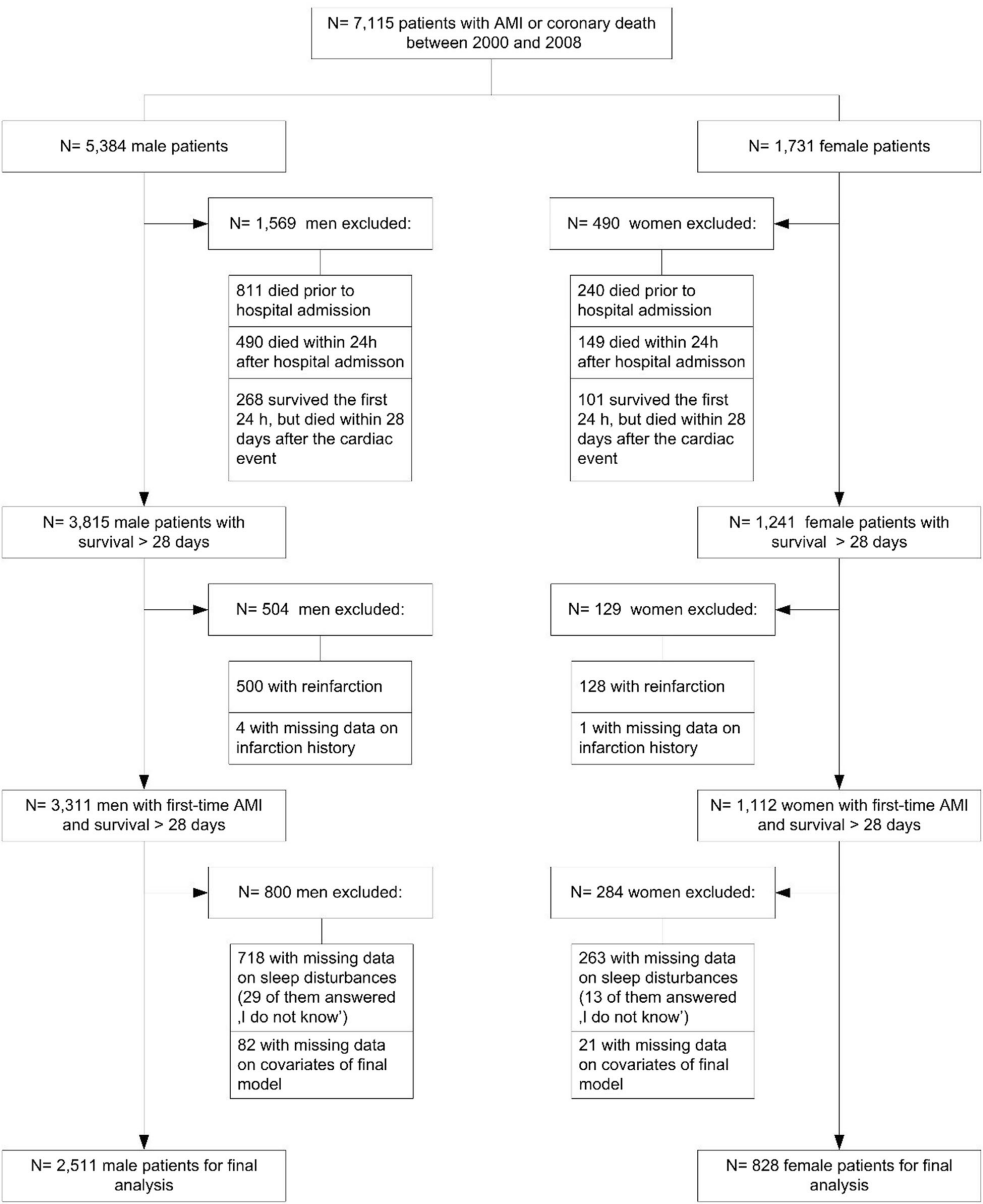
Flow chart of study sample selection

Based on a total sample of 7115 registered patients between January 1, 2000 and December 31, 2008, our study question was confined to those who had first-time AMI and survived for at least 28 days after the event (*n* = 4423). We further excluded those without information on sleep disturbances (*n* = 981) as well as patients with missing data on relevant covariables to be included in final regression models (*n* = 103), leaving 2511 men and 828 women eligible for the analyses of long-term survival (Fig. 1). Patients with missing values on sleep disturbances had a worse prognosis compared to those with available data (crude Hazard Ratio (HR) for long-term mortality 2.72; 95% confidence interval (CI): 2.29–3.22 (men) and HR 2.14; CI 1.62–2.83 (women)). In most cases, sleep information was lacking because no interview was conducted (96.6%).

Information on sleep disturbances was collected by one questionnaire item. ‘How often did you suffer from sleep disturbances within the last 4 weeks?’ could be answered by the response categories ‘1: never’, ‘2: less than once per week’, ‘3: once per week’, ‘4: more than once per week’, ‘5: every night’, ‘6: I do not know’. Patients who indicated the latter (*n* = 42) were treated as cases with missing data. For the 3-categorical primary independent variable ‘frequency of sleep disturbances’, we summarized answers to ‘never’ (original category 1), ‘sometimes’ (original categories 2–4) and ‘every night’ (original category 5) assuming that patients suffering not at all (1) or nightly (5) would rate themselves rather accurately.

The following covariables were captured and considered for analysis: patients were asked whether they were employed (currently, formerly, never), whether they were married (yes/no) and whether they smoked (currently, formerly, never). The highest acquired school-leaving qualification was captured to evaluate educational level (according to the German school system: 1 = lower secondary school; 2 = secondary school; 3 = high school diploma; 4 = university degree; 5 = other qualification; 6 = refused to reply). This information was dichotomized for analysis as ‘low education (< 9 years)’ which corresponds to response category 1 (yes/no). Body mass index (BMI) was obtained by assessment of height and weight during the hospital stay and adiposity was defined as BMI > 30 kg/m^2^ (yes/no). While the history of stroke (yes/no) was only determined by self-report, medical history of angina pectoris, hypertension, hyperlipidemia or diabetes (yes/no) was considered if confirmed by chart review. The type of AMI was defined as ST-segment elevation MI (STEMI), Non-ST-segment elevation MI (NSTEMI), bundle branch block, or non-classifiable/missing. The latter two categories were summarized to ‘non-classifiable’ for analysis. Regarding primary care, revascularization therapy (yes/no) was defined as having received thrombolysis, percutaneous transluminal coronary angioplasty with or without stenting or coronary artery bypass surgery. A combination of the following four evidence based medications is strongly recommended as standard of care after AMI with reported benefit on long-term survival: anti-platelet agents, beta-blockers, statins and angiotensin-converting enzyme inhibitors or angiotensin-receptor blockers [17]. Thus, we summarized data on medication at hospital discharge to treatment with all 4 evidence based medications (yes/no).

The outcome of interest was all-cause mortality, assessed by checking the vital status of all recorded persons in the MI registry. Local health departments inside and additionally outside the study region were involved in the mortality follow-up assessment, which takes place about every 5 years, in order to ascertain the survival status of all participants including those who had moved out of the study area. Follow-up for this study ended in December 2011. Survival time was measured in days and defined from day of the acute event to the day of death. Patients who were still alive at the end of the follow-up period were censored.

Analyses were done separately for men and women due to differences in CVD risk profile and outcome [18] and differences in both their reported [3] and objective sleep habits [19, 20], comparable to previous investigations [14]. The primary independent variable was cross-tabulated with potential covariables. Categorical ones were presented as absolute numbers and percentage, and evaluated for differences in frequency distribution with χ^2^-test. Continuous data was expressed as median with interquartile range (IQR) and compared using Kruskal-Wallis test as non-parametric equivalent to one-way analysis of variance.

Differences in survival time were determined by Log-Rank test for all categorical variables or simple Cox regression models for continuous age and BMI, respectively.

Except for age, which was a priori determined to be forced in regression models, only those variables were considered for subsequent regression modeling which were significantly associated with either sleep disturbances or long-term survival in sex-specific comparisons.

Kaplan-Meier-curves were generated for the primary independent variable. Differences in survival time between the three strata of the primary independent variable were determined by log rank test. To estimate the magnitude of impact of sleep disturbances on all-cause mortality, several Cox proportional hazard regression models were fitted. Hazard ratios were presented with 95%- confidence intervals and patients never having suffered from sleep disturbances within 4 weeks prior to AMI as reference category. The overall influence of the 3-categorical primary independent variable was evaluated using Likelihood-Ratio tests. First, univariable regression models were calculated for men and women to assess the crude association followed by age-adjusted models. Minimally adjusted models were then individually expanded for covariables sets from the domains socio-demographic/lifestyle, comorbidities and clinical aspects. Finally, a full model - identical for both sexes - was fitted containing all covariables with either significant association in the male or female data set. To control for possible cohort effects, the year of AMI was also included. The potential mediating role of hypertension and diabetes [21, 22] on the association of sleep disturbances and long-time mortality was considered by refitting the full models without these variables.

Proportional hazards assumption was checked graphically (parallel lines of log (−log(survival)) versus log of survival times) for all variables. In case of violating the proportional hazards requirement, a time-dependent variable was created for the respective variable and tested in a bivariate cox regression model for statistical significance. Solely the interaction terms for age and BMI with log (survival) in the female group were statistically significant. Since HRs for the primary independent variable hardly changed when time-dependent variables were included, we decided to report the results for the simple models without interaction terms.

Interaction effects of age with sleep disturbances were tested in bivariate and full models, but failed to reach statistical significance.

To account for the arbitrary categorization of the primary independent variable, sensitivity analyses were performed with the following two alternative categorizations in age-adjusted and full models: 1) another 3-categorical allocation (never [original category 1], occasionally [original categories 2, 3], frequently [original categories 4, 5]) and 2) a binary classification (any sleep disturbances in the prior 4 weeks yes [1]/ no [2–5]). In further sensitivity analyses, we restricted the male study population to those patients with normal weight (BMI ≥18.5 and < 25 kg/m^2^), in order to consider potential confounding through obstructive sleep apnea, which occurs more often in overweight men [23]. Finally, crude and age-adjusted models were refitted including also patients with missing data on any of the covariates of the full model.

For all investigations, a significance level of 5% was applied. Data management and analysis were performed using SAS version 9.2 (SAS Institute Inc., Cary, North Carolina).

## Results

The study sample consisted of 2511 male and 828 female patients. With a median age of 65 years (IQR: 12) women were older than men (60 (IQR: 15)) and more often diagnosed with hypertension and diabetes.

Baseline socio-demographic data, lifestyle factors, comorbidities, AMI and treatment characteristics of male and female patients stratified by frequency of sleep disturbances are presented in Table 1. Almost 50% of female patients indicated having been affected by sleep disturbances anytime (30.3% sometimes and 18.1% nightly) whereas about 30% of the male sample reported sleep disturbances preceding AMI (22.7 and 9.6%, respectively). These numbers changed in the course of the study period starting with a proportion of 35% of women in the year 2000 up to almost 60% 8 years later. An even stronger increase from early 18% to later 48% reporting any sleep disturbance was observed for men. Median age did not significantly vary across categories of sleep disturbances for both sexes. Overall, none of the analyzed variables were significantly associated with frequency of sleep disturbances in female patients, but for men some differences were detected. Among others, BMI of men was slightly, but significantly, different in the sleep categories with the highest median value in the ‘nightly’ group (27.7, IQR: 5.3). The proportion of male hypertensive patients increased with frequency of sleep complaints from 71.8 to 80.1%. The same trend applied to patients diagnosed with angina pectoris. Regarding revascularization therapy, male patients in the bottom as well as in the upper sleep category more often received any of the mentioned treatments.

**Table 1:** Sample characteristics for male and female AMI patients stratified by frequency of sleep disturbances

	Men (*n* = 2511)				Women (*n* = 828)			
	SLEEP DISTURBANCES			*p*-value^a^	SLEEP DISTURBANCES			*p*-value^a^
	never *n* = 1699	sometimes *n* = 571	nightly *n* = 241		never *n* = 427	sometimes *n* = 251	nightly *n* = 150	
Socio-demographic Data								
Age, years [median (IQR)]	60 (15)	60 (15)	60 (14)	0.7213	65 (13)	65 (13)	65 (12)	0.8775
Low Education, < 9 years	1186 (69.8)	407 (71.3)	170 (70.5)	0.7958	351 (82.2)	202 (80.8)	113 (75.3)	0.1894
Married	1378 (81.1)	435 (76.2)	190 (78.8)	0.0375	258 (60.42)	147 (58.57)	91 (60.67)	0.8732
Currently employed	788 (46.4)	243 (42.6)	99 (41.1)	0.1236	98 (22.95)	50 (19.92)	26 (17.33)	0.3057
Lifestyle factors								
BMI, kg/m^2^ [median (IQR)]	27.1 (4.8)	27.4 (4.7)	27.7 (5.3)	0.0370	26.8 (7.4)	27.5 (7.1)	27.7 (7.0)	0.5334
Adiposity, BMI > 30 kg/m^2^	376 (22.1)	141 (24.7)	69 (28.6)	0.0566	134 (31.4)	86 (34.3)	50 (33.3)	0.7258
Ever Smoker	1280 (75.3)	402 (70.4)	187 (77.6)	0.0322	209 (49.0)	112 (44.6)	76 (50.7)	0.4216
Comorbidities								
Angina	190 (11.2)	104 (18.2)	52 (21.6)	< 0.0001	69 (16.16)	30 (11.95)	31 (20.67)	0.0631
Hypertension	1219 (71.8)	438 (76.7)	193 (80.1)	0.0040	350 (82.0)	206 (82.1)	129 (86.0)	0.5034
Dyslipidemia	1220 (71.8)	394 (69.0)	172 (71.4)	0.4393	310 (72.6)	187 (74.5)	119 (79.3)	0.2665
Diabetes	443 (26.1)	143 (25.0)	59 (24.5)	0.8022	137 (32.1)	82 (32.7)	53 (35.3)	0.7647
Stroke	75 (4.4)	29 (5.1)	15 (6.2)	0.4232	28 (6.6)	15 (6.0)	14 (9.3)	0.4070
Clinical aspects								
Type of Infarction								
*STEMI*	710 (41.8)	204 (35.7)	86 (35.7)	0.0756	180 (42.2)	87 (34.7)	64 (42.7)	0.2622
*NSTEMI*	872 (51.3)	323 (56.6)	137 (56.9)		229 (53.6)	149 (59.4)	81 (54.0)	
*Bundle Branch Block/non-classifiable*	117 (6.9)	44 (7.7)	18 (7.5)		18 (4.2)	15 (6.0)	5 (3.3)	
Revascularization therapy	1521 (89.5)	490 (85.8)	221 (91.7)	0.0175	338 (79.2)	195 (77.7)	111 (74.0)	0.4254
4 EBM at discharge	1221 (71.9)	418 (73.2)	183 (75.9)	0.3852	285 (66.7)	180 (71.7)	111 (74.0)	0.1699
Mortality								
Deceased during follow-up	206 (12.1)	87 (15.2)	25 (10.4)	0.0818	71 (16.6)	37 (14.7)	23 (15.3)	0.7964

Data is presented as absolute number (%) unless otherwise indicated.

*AMI* acute myocardial infarction, *BMI* Body Mass Index, *EBM* evidence based medication, *IQR* interquartile range, *NSTEMI* Non-ST-segment elevation myocardial infarction, *STEMI* ST-segment elevation myocardial infarction

^a^*p*-values are obtained from χ^2^-tests for categorical data or from Kruksal-Wallis test for continuous data (age and BMI)

**Fig. 2:**
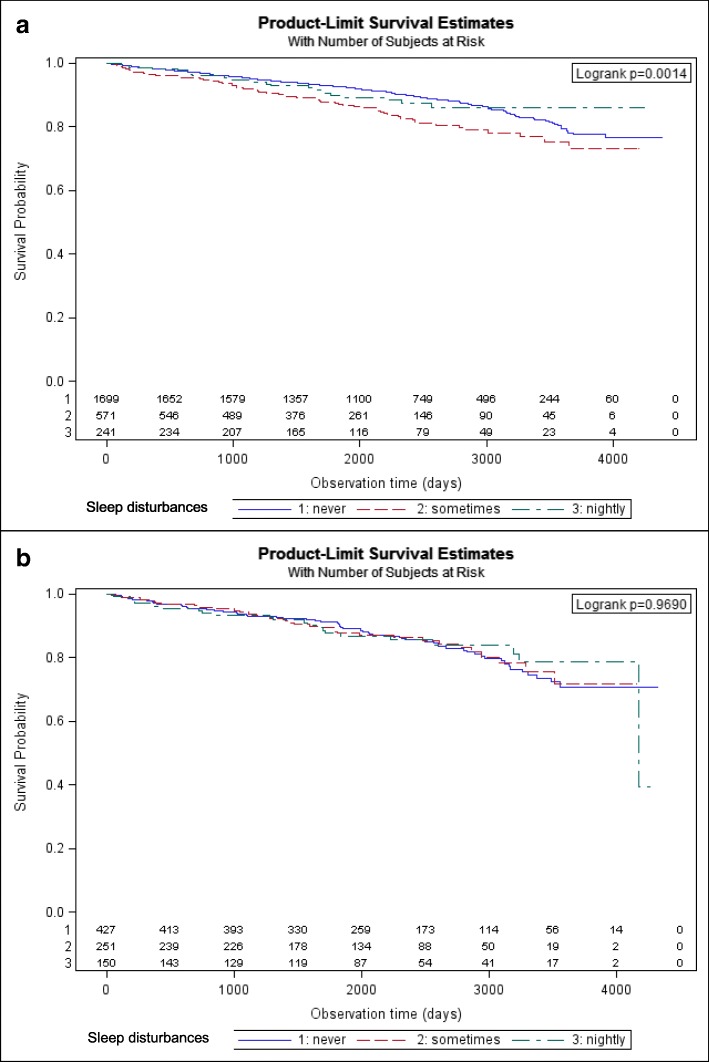
12-year survival curves for male (**a**) and female (**b**) AMI patients stratified by frequency of sleep disturbances. Footnote: “Differences in survival time between the three strata of the primary independent variable were determined by log rank test.”

Median follow-up time was 6.1 years in men and women (IQR: 4.1). Overall, women had a greater long-term mortality rate than men (26 vs. 21 deaths per 1000 person years, respectively). For men, the highest mortality rate was observed for patients who sometimes suffered from sleep disturbances (28 deaths per 1000 person years vs. 19 and 18 deaths per 1000 person years for the bottom and upper category, respectively) while the mortality rate for females did not differ between categories. Kaplan-Meier survival curves visualizing these tendencies in Fig. 2 demonstrated significant differences in survival according to frequency of sleep disturbances for men (Log-Rank, *p* = 0.0014), but not for women (Log-Rank, *p* = 0.9690).

Results from Cox regression analyses are presented in Table 2. The age-adjusted model for men revealed a 56% increased all-cause mortality risk for patients who sometimes suffered from sleep disturbances compared to those reporting no sleep disturbances (HR 1.56; 95%-CI 1.21–2.00) whereas male patients who indicated having had disturbed sleep every night did not show an elevated relative risk (HR 1.00; 95%-CI 0.66–1.51). By separately adding covariables sets from the domains ‘socio-demographic/lifestyle’, ‘comorbidities’, and ‘clinical aspects’ to the age-adjusted model, HRs hardly changed. The decrease of HR [sometimes vs. never] by 5.8% was greatest for the model ‘Clinical’ and mainly explained by revascularization treatment. The full model finally attenuated the long-term mortality risk for the second category to 1.40 (HR, 95%-CI 1.08–1.81). However, the increase remained significant as well as the overall impact of sleep disturbances (Likelihood-Ratio Test *p* = 0.0370).

**Table 2:** Association of frequency of sleep disturbances within 4 weeks prior to AMI (categorization 1) and long-term mortality in male and female patients

		Men (*n* = 2511; 318 deaths)			Women (*n* = 828; 131 deaths)		
		HR	[95% CI]	*p*-value^a^	HR	[95% CI]	*p*-value^a^
	Frequency of sleep disturbances						
Unadjusted model	never	1	Ref.	0.0024	1	Ref.	0.9686
	sometimes	1.58	[1.23–2.03]		1.00	[0.67–1.49]	
	nightly	1.00	[0.66–1.52]		0.94	[0.59–1.51]	
Age-adjusted model	never	1	Ref.	0.0032	1	Ref.	0.9773
	sometimes	1.56	[1.21–2.00]		0.97	[0.65–1.44]	
	nightly	1.00	[0.66–1.51]		0.96	[0.60–1.53]	
Model Lifestyle^b^	never	1	Ref.	0.0038	1	Ref.	0.9665
	sometimes	1.55	[1.20–1.99]		0.96	[0.64–1.43]	
	nightly	0.99	[0.65–1.50]		0.95	[0.59–1.52]	
Model Comorbidities^c^	never	1	Ref.	0.0060	1	Ref.	0.9556
	sometimes	1.50	[1.16–1.93]		0.98	[0.66–1.47]	
	nightly	0.92	[0.60–1.39]		0.93	[0.58–1.49]	
Model Clinical^d^	never	1	Ref.	0.0150	1	Ref.	0.9899
	sometimes	1.47	[1.14–1.89]		0.97	[0.65–1.46]	
	nightly	1.02	[0.67–1.54]		0.97	[0.61–1.57]	
Full model^e^	never	1	Ref.	0.0370	1	Ref.	0.9055
	sometimes	1.40	[1.08–1.81]		0.99	[0.66–1.49]	
	nightly	0.95	[0.62–1.45]		0.90	[0.55–1.46]	

AMI = acute myocardial infarction, CI = confidence interval, HR = Hazard Ratio

^**a**^*p*-value obtained from Likelihood-Ratio test for overall significance of 3-categorical variable ‘frequency of sleep disturbances’ in the respective model

^**b**^MEN: adjusted for age (cont.), married, low education (< 9 years), employment status, smoking ever and BMI (cont)

WOMEN: adjusted for age (cont.), married and employment status

^**c**^MEN: adjusted for age (cont.), hypertension, angina, diabetes, dyslipidemia and stroke

WOMEN: adjusted for age, diabetes and stroke

^**d**^MEN: adjusted for age (cont.), type of infarction, any revascularization treatment (coronary artery bypass surgery, percutaneous coronary intervention (PCI) with/without stenting, or thrombolysis), all four evidence based medications at discharge (antiplatelet agents, beta-blockers, ACEIs/ARBs (Angiotensin-converting enzyme inhibitors/Angiotensin receptor blockers), statins) and year of MI

WOMEN: adjusted for age (cont.), type of infarction, any revascularization treatment and all four evidence based medications at discharge

^**e**^MEN AND WOMEN: adjusted for age (cont.), low education (< 9 years), married, employment status, ever smoking, BMI (cont.), hypertension, angina, diabetes, dyslipidemia, stroke, type of infarction, any revascularization treatment, all four evidence based medications at discharge and year of MI

For women, a relation between sleep disturbances prior to AMI and long-term mortality was detected neither in the age-adjusted or subset models nor in the full model controlled for the same confounders as used in the male model.

When the potential mediator variables hypertension and diabetes were removed individually and together from the full model, HR estimates for sleep disturbances did not change remarkably both for men and women (data not shown).Table 3 summarizes the distribution of the study sample depending on the respective categorization of frequency of sleep disturbances. As sensitivity analysis we refitted the age-adjusted and full model for males and females with alternative categorizations of the primary independent variable. Results of the regression models are shown in Table 4. For the other 3-level variable (categorization 2), the influence of sleep disturbances on long-term mortality in male AMI patients lost overall significance (Likelihood-Ratio Test *p* = 0.1254). Likewise, the binary comparison failed to reach statistical significance (HR 1.26; 95% CI 1.00–1.60, *p* = 0.0543). Again, for women no associations were observed.

**Table 3:** Distribution of patients and events according to different categorizations of the primary independent variable

Frequency of sleep disturbances within 4 weeks prior to AMI		Men (*n* = 2511)		Women (*n* = 828)	
		No. per category (%)	No. of deaths	No. per category (%)	No. of deaths
Original response	never	1699 (67.7)	206	427 (51.6)	71
	< 1 per week	247 (9.8)	40	82 (9.9)	14
	once per week	67 (2.7)	10	31 (3.7)	7
	> 1 per week	257 (10.2)	37	138 (16.7)	16
	nightly	241 (9.6)	25	150 (18.1)	23
Categorization 1	never	1699 (67.7)	206	427 (516)	71
	sometimes	571 (22.7)	87	251 (30.3)	37
	nightly	241 (9.6)	25	150 (18.1)	23
Categorization 2	never	1699 (67.7)	206	427 (51.6)	71
	occasionally	314 (12.5)	50	113 (13.7)	21
	frequently	498 (19.8)	62	288 (34.8)	39
Binary (any time)	no	1699 (67.7)	206	427 (51.6)	71
	yes	812 (32.3)	112	401 (48.4)	60

*AMI* acute myocardial infarction

**Table 4:** Association of frequency of sleep disturbances within 4 weeks prior to AMI (categorization 2 and binary split) and long-term mortality in male and female patients

		Men (*n* = 2511; 318 deaths)			Women (*n* = 828; 131 deaths)		
		HR	[95% CI]	*p*-value^a^	HR	[95% CI]	*p*-value^a^
	Frequency of sleep disturbances						
Age-adjusted model	never	1	Ref.	0.0183	1	Ref.	0.7162
	occasionally	1.52	[1.11–2.07]		1.12	[0.68–1.82]	
	frequently	1.29	[0.97–1.72]		0.90	[0.61–1.33]	
Full model^b^	never	1	Ref.	0.1256	1	Ref.	0.7163
	occasionally	1.37	[1.00–1.87]		1.10	[0.67–1.82]	
	frequently	1.19	[0.89–1.59]		0.89	[0.59–1.33]	
	Sleep disturbances at any time						
Age-adjusted model	no	1	Ref.		1	Ref.	
	yes	1.38	[1.10–1.74]	0.0070	0.96	[0.68–1.36]	0.8330
Full model^b^	no	1	Ref.		1	Ref.	
	yes	1.26	[1.00–1.60]	0.0571	0.95	[0.67–1.36]	0.7854

*AMI* acute myocardial infarction, *CI* confidence interval, *HR* Hazard Ratio

^a^p-value obtained from Likelihood Ratio test for overall significance of ‘frequency of sleep disturbances’ in the respective model

^b^adjusted for age (cont.), low education (< 9 years), married, employment status, ever smoking, BMI (cont.), hypertension, angina, diabetes, dyslipidemia, stroke, type of infarction, any revascularization treatment (coronary artery bypass surgery, percutaneous coronary intervention (PCI) with/without stenting, or thrombolysis), all four evidence based medications at discharge (antiplatelet agents, beta-blockers, ACEIs/ARBs (Angiotensin-converting enzyme inhibitors/Angiotensin receptor blockers), statins) and year of MI

The association of sleep disturbances and all-cause mortality was not seen any more in the full model restricted to the 629 male patients with normal weight (HR [sometimes vs. never] 1.16, 95%-CI 0.72–1.88 and HR [nightly vs. never] 1.48, 95%-CI 0.76–2.88).

In a final sensitivity analysis, adding the patients with missing data on any covariables of the full model (additionally 82 males and 21 females), no notable changes regarding the unadjusted and age-adjusted HRs resulted (data not shown).

## Discussion

In our study, we found a 40% increased long-term mortality risk in the fully adjusted model for male AMI patients who sometimes, i.e. up to several times a week, suffered from sleep disturbances within 4 weeks before the acute event. For men with nightly sleep disturbances and for women, however, no associations between sleep disturbances and long-term mortality were seen in our data.

Overall, sleep disturbances were reported by 36.3% of our total sample of AMI patients, which is somewhat higher than referred for the general population [2]. The proportion was considerably larger in women than in men (48.4% vs. 32.3%). This was not surprising since women are known to be more frequently affected by insomnia symptoms due to hormonal and physiological differences as well as psychosocial factors [24]. Depression and anxiety disorders which are almost twice as prevalent in women compared to men [25] and are associated with sleep disturbances [26], represent a relevant factor regarding the female predominance in insomnia symptoms [20]. Moreover, prevalence of insomnia was shown to increase in postmenopausal women compared to premenopausal women [27]. With a median age of 65 years in our female study sample, the majority of women could be assumed to be in postmenopausal phase. Finally, sleep disturbances increase with age [2]. This trend might also contribute to the difference in proportions of reported sleep disturbances in our study, as women were significantly older in the analyzed data.

Overall, unadjusted percentages of both men and women who reported sleep disturbances in our study clearly increased within the study period from the year 2000 to 2008 by factor 2.7 and 1.7 respectively. Previous epidemiological studies in several countries presented increasing prevalence of sleep complaints on population-level in the last decades [28]. Among others, the demographic change and growing health problems such as the obesity epidemic were considered as reasons for this development as well as altering occupational requirements such as shift-work and increased work-load [1, 28]. Nevertheless, this trend might be at least in part influenced by a sort of reporting bias due to growing awareness for sleep problems.

Referring to the International Classification of Sleep Disorders – 3rd edition [29] (ICSD-3) and the Diagnostic and Statistical Manual of Mental Disorders – 5th edition [30] (DSM-5) criteria for insomnia disorder, many studies applied a frequency of at least three times per week to rate sleep disturbances as symptomatic [2]. Transferred to our data, 23.5% reported to have suffered from sleep disturbances several times a week or nightly, which corresponds to category ‘frequently’ in categorization 2 (19.8% of men and 34.8% of women, respectively). These numbers were similar to those stated in two previous studies on the association of sleep disturbances prior to AMI with long-term outcomes of patients [13, 14], but the assessment and definition of sleep disturbances varied.

In the cohort study by Condén & Rosenblad [13] 23.9% of 732 Swedish AMI patients, recruited between 2006 and 2011, were classified as having insomnia, which was defined in the study as difficulty falling asleep without considering the frequency of occurrence (19.7% of men and 32.7% of women, respectively). Further, the corresponding question was not limited to the period prior to the cardiovascular event and hence might also have captured cases which had problems with initiating sleep as a result of the MI. This could have influenced the survival prognosis in the group of insomnia patients who had an almost 60% elevated, multivariable adjusted, all cause-mortality risk in the period after the first 2 years of follow-up (mean follow-up time 6.0 years) compared to patients not having suffered from difficulties falling asleep. Thus, the relative long-term mortality risk for male and female insomnia patients in Condén & Rosenblad [13] was higher than the fully adjusted HR that we found for men who had sometimes suffered from sleep disturbances (HR 1.40, 95%-CI 1.08–1.81) or men who reported sleep disturbances at any time within 4 weeks prior to AMI (HR 1.26; 95%-CI 1.00–1.60).

In the second previous study investigating long-term outcomes after AMI by Clark et al. [14] based on the Stockholm Heart Epidemiology Program (SHEEP), sleep impairment was assessed in more detail for 1089 male and 499 female patients with first-time AMI between 1992 and 1994. One of four sleep-related aspects was disturbed sleep, which comprised items on general sleep quality, difficulty falling asleep, repeated awakenings, disturbed/uneasy sleep and premature awakenings. More than 20% of the male patients and about one third of the females reported to have experienced disturbed sleep. Unlike in our interrogation, patients were asked for sleep impairment within 1 year prior to initial AMI. Hence, reported sleep disturbances might be more likely associated also with other health problems besides MI. A significantly increased risk for incidence of or death from cardiovascular events (distinguishing AMI [age-adjusted HR 1.73; 95%-CI 1.03–2.91], stroke [multiple adjusted HR 2.61; 95%-CI 1.19–5.76] or heart failure [multiple adjusted HR 2.43, 95%-CI 1.18–4.97]) within a 10-year follow-up was revealed for women but not for men with disturbed sleep at least sometimes a week compared to persons with less frequent or no complaints. As the endpoint of our study was all-cause mortality and we were not able to consider recurrent cardiovascular events after MI and cause-specific mortality, our results were not comparable to the above mentioned study.

Within the framework of topic-related studies, the results of our study therefore had to stand for themselves. We found an association of sometimes experienced sleep disturbances and long-term all-cause mortality in male AMI patients, but not in female patients. Reasons for this sex difference can only be speculative.

Firstly, sex differences in sleep architecture and in implications of sleep disturbances might contribute to the present results. Cross-sectional analyses of healthy participants showed that the percentage of deeper slow wave stage, which is important for regeneration during sleep [31], was significantly lower in men across all age groups and decreased in men getting older but not in women [19, 32]. However, sleep continuity and architecture were shown to change when sleep complaints became pathological. A recent meta-analysis showed a significant reduction of slow wave and rapid eye movement sleep in patients with primary insomnia compared to a good sleeper control group [33]. How far the above-mentioned sex differences hold also for people complaining about sleep disturbances needs to be examined. Secondly, one study found that healthy women were more resilient to the consequences of elevated circulating inflammatory markers induced by sleep disturbances than healthy men [34]. If these observations apply in a similar manner to a MI population, women might cope better with the consequences of sleep disturbances and thus repel a potential adverse impact on long-term survival.

Thirdly, though women across all age strata subjectively reported more sleep complaints than men [3], some studies revealed no relative differences in polysomnography measurements or even better objective sleep quality for women [20, 24, 32]. Hence, we cannot fully rule out misclassification of female patients as a consequence of misperception of sleep-state to cause sex differences in long-term prognosis in our study and to obscure a potential association in women.

Contrary to what one might expect, we also did not detect an increased mortality risk for patients who suffered nightly from sleep disturbances within 4 weeks preceding the AMI. This may be a result of fewer patients in this category leading to insufficient power to reveal a possible association. Another reason could be a change in individual frequency of sleep disturbances during the follow-up period, particularly as a consequence of the AMI and its severity. As sleep disturbances were assessed only once in our registry, we could not investigate this aspect in our analysis. Adding those patients who suffered more than once per week from disturbed sleep to the nightly group (yielding category ‘frequently’ of categorization 2), did not reveal a significant increase in mortality risk either. These sensitivity analyses, including the binary comparison, question the robustness of the observed association for male patients found for categorization 1 and stress the need for further investigations. Another reason for our unexpected non-significant result observed in patients who suffered nightly from sleep disturbances might be driven by most resent literature on the role of chronic obstructive sleep apnea as a pre-conditioning stimulus that may have cardioprotective effects in some AMI patients [11]. Shah et al. [11] found a high prevalence (77%) of sleep disordered breathing among patients with AMI and observed that patients with obstructive sleep apnea (35%) develop a less severe cardiac lesion than those without obstructive sleep apnea. These findings question the previous literature that found obstructive sleep apnea to be an independent risk factor for fatal and non-fatal CVD. Nightly sleep disorder caused by non-treated obstructive sleep apnea may have influenced our results and warrants further investigation.

As observational studies still varied widely in the definition of insomnia and other sleep complaints, future research should draw profit from improvements and assimilation of existing diagnostic classification systems of sleep disorders and research diagnostic criteria for insomnia [29, 30]. For this purpose, a harmonization of the assessment of sleep disturbances in epidemiological research would be helpful to yield comparable results and hence to contribute to evident findings. Based on the recently revised ICSD − 3 and DSM-5 criteria for insomnia, a commonly accepted approach to assess sleep disturbances on a population-level should at least specify the kind of symptoms to be captured, their frequency in a defined time period as well as their severity with regard to daytime consequences.

To the best of our knowledge, the present study was the first to examine associations of patient-reported sleep disturbances shortly before AMI and long-term all-cause mortality separately for men and women using data from the percutaneous coronary intervention era.

A further strength of our study is the large sample from a population-based MI registry with high quality and validation standards contributing to the generalizability of our results.

Finally, we were able to adjust for a number of potential confounders, comprising lifestyle factors, comorbidities and clinical aspects.

Nevertheless, some important potential confounders were lacking. First, we were not able to adjust for depressive symptoms though depression is known to be associated with sleep disturbances [26] as well as cardiovascular outcomes and mortality [35].

Likewise, our study did not control for obstructive sleep apnea which may have a confounding influence on the relation between sleep disturbances and long-term mortality after MI [7, 10]. To account for this issue we did a sensitivity analysis in normal weight male patients, as obstructive sleep apnea is more frequent in overweight men [23]. Sleep disturbances were not significant anymore in the full model, but estimates resulted from a markedly smaller patient group with only 11 events in the category of nightly sleep disturbances and have to be treated with caution. However, the aspect of weight was not disregarded in the primary analysis as regression models are adjusted for BMI.

Finally, data on sleep disturbances was based on self-report in this study and resulted from only one questionnaire item. However, in clinical practice no objective sleep data will be available either and physicians have to rely on compact patient information. Nevertheless, sleep disturbances in our study were uniquely assessed right after the AMI and we did not know about frequency of sleep disturbances in the follow-up period.

## Conclusion

In summary, our data indicate that sleep disturbances were more frequently reported in AMI patients over time with a higher increasing prevalence observed in males. We found an association of sometimes experienced sleep disturbances and long-term all-cause mortality in male but not female AMI patients. In patients who reported nightly sleep disturbances, we did not found a significant association. This unexpected latter result and the sex-differences observed in our study need further investigations considering the diagnosis and treatment of commonly reported obstructive sleep apnea in AMI patients.

## Abbreviations

AMI: Acute myocardial infarction
BMI: Body mass index
CI: Confidence interval
CVD: Cardiovascular disease
DSM: Diagnostic and statistical manual of mental disorders
HR: Hazard ratio
ICSD: International classification of sleep disorders
IQR: Interquartile range
KORA: Cooperative health research in the Augsburg region
LR: Likelihood ratio
MI: Myocardial infarction
MONICA: Monitoring trends and determinants in cardiovascular disease
NSTEMI: Non-ST segment elevation myocardial infarction
STEMI: ST-Segment elevation myocardial infarction
